# Supplementary material for: MSX1 Regulates Goat Endometrial Function by Altering the Plasma Membrane Transformation of Endometrial Epithelium Cells during Early Pregnancy
Source: Int J Mol Sci. 2023 Feb 18;24(4):4121. doi: 10.3390/ijms24044121 (PMC9960665; doi:10.3390/ijms24044121)
Supplement: Supplementary file 1 [file ijms-24-04121-s001.zip › ijms-2185284-supplementary.pdf]

Supplemental table

Table S1. Primer pairs used for real time quantitative PCR

Gene	Sequences (5`-3`)	Accession number
GAPDH	Forward: GATGGTGAAGGTCGGAGTGAAC	XM_005680968.3
	Reverse: GTCATTGATGGCGACGATGT	
Msx1	Forward: TGCCTCCTGTCCTAACACCT	XM_018049722
	Reverse: CTGCCTCTCCTGCAGTTCTC	
ZO-1	Forward: CCTGACGGTTGGTCTTTTGC	XM_018066114
	Reverse: ACAGAAACACAGTTTGCGCC	
α-PKC	Forward: TCCATTTACCGACGAGGTGC	XM_005675332
	Reverse: CCTGTTGAAACGCTTGGCTT	
Par3	Forward: TCCGCCAGGTTTCTTCAGTC	XM_005692148
	Reverse: CGGAACCATGCTTATGCAGC	
CDH2	Forward: TACAGTGCAGTCTTGTCCCG	XM_018039719
	Reverse: GCTCGCTGCTCTCATACTGT	
SCRIB	Forward: GGAGGCAACGATCTGGAAGT	XM_018045180
	Reverse: CTGATAGCTGGTTCCGGTCC	
Lgl2	Forward: TCCTTCACAGGCTTCGTC	XM_018063810
	Reverse: CTGGCTCATCCATTCTCC	
PTGS1	Forward: TCACAGTGCGTTCCAACCTTATC	[27]
	Reverse: ACGGAGGGCAGAATGCGAGTA	
PTGS2	Forward: GAGTGTAGGATTCGACCAGTAT	[27]
	Reverse: CCTTGAAGTGGGTAAGTATGTAG	
PTGES	Forward: CATCAAAATGTACGCGGTGGC	[27]
	Reverse: GTCCTCGGGGTTGGCAAAAGC	
PGFS	Forward: TGGAGGACCCAGTTCTTTGTG	[27]
	Reverse: TACCTGATAGCGAAGGGCAAC	
ISG15	Forward: GGTGAGGAACGACAAGGGTC	[35]
	Reverse: CAGAATTGGTCCGCTTGCAC	
RSAD2	Forward: GGTTTTCTTATTTTCTGCCTTAT	[35]
	Reverse: ATCCATTACTGATCTCGATGC	
CXCL10	Forward: TGCTTGGTGCCCGAGTCTAAC	[35]
	Reverse: TCCGCCCATTTCTACAGTTCA	

Table S2. Short hairpin interfering RNA (shRNA) inserts

shRNA	**Sequence (5'-3')**
ShRNA-Msx1 F	GATCCGCTACAGCATGTACCACCT CTCGAG AGGTGGTACATGCTGTAGCTTTTTG
ShRNA-Msx1 R	AATTCAAAAAGCTACAGCATGTACCACCTCTCGAGAGGTGGTACATGCTGTAGCG
ShN F	GATCCTTCTCCGAACGTGTCACGTTTCAAGAGAACGTGACACGTTCGGAGAATTTTTTG
ShN R	AATTCAAAAAATTCTCCGAACGTGTCACGTTCTCTTGAAACGTGACACGTTCGGAGAAG

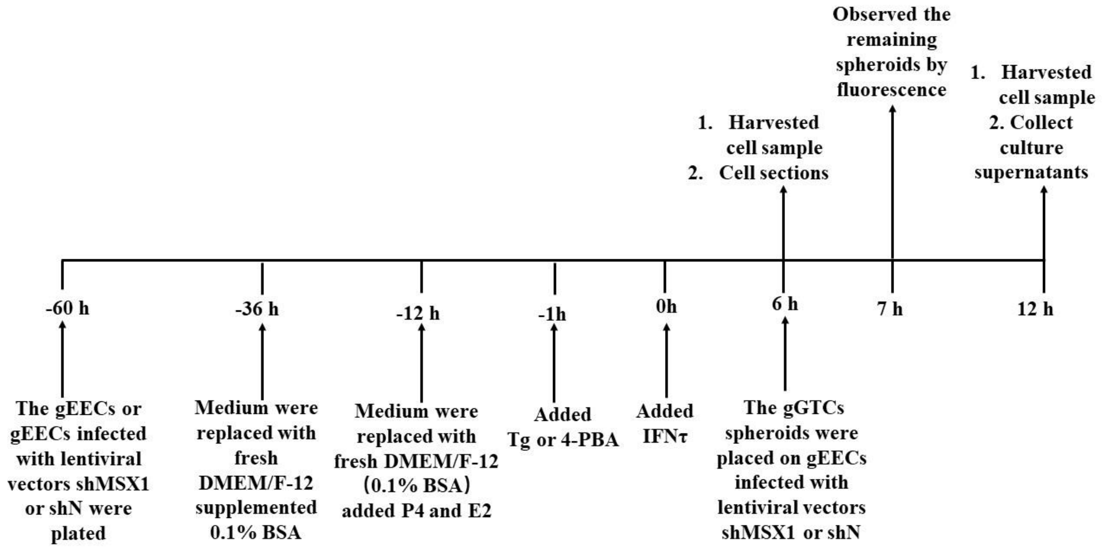

Figure S1. Schedule of the gEECs treatment. At -60 h, the gEECs or gEECs infected with lentiviral vectors shMSX1 or shN were plated. From -36 to -12 h, gEECs was grown in fresh DMEM/F-12 plus 0.1 % BSA; -12 to 0 h is the stage in which maternally derived P4 and E2 were added; 0-12 h is the stage in which conceptus-derived IFNτ was added. 0 h is the time of IFNτ addition.

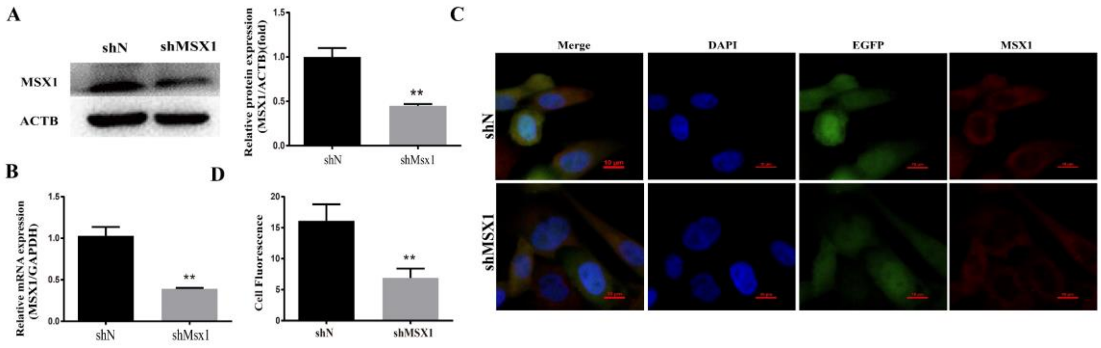

Figure S2. Interference efficiency of shMSX1 vectors. (A) The representative images and quantitative analysis of MSX1 protein level. (B) The quantitative analysis of MSX1 mRNA level. (C-D) The representative confocal microscopeimages and quantitative analysis of MSX1 expression in shN group and shMSX1group. “*” as P<0.05; “**” as P<0.01.

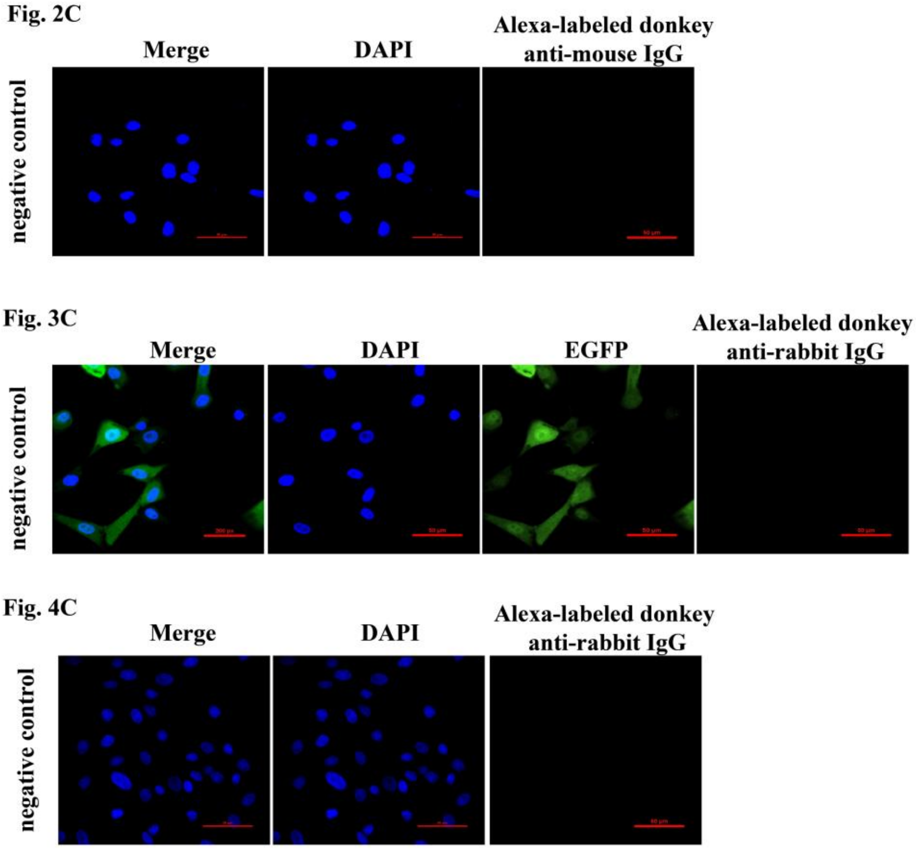

Figure S3. The amplification curve and melt curve of primers.

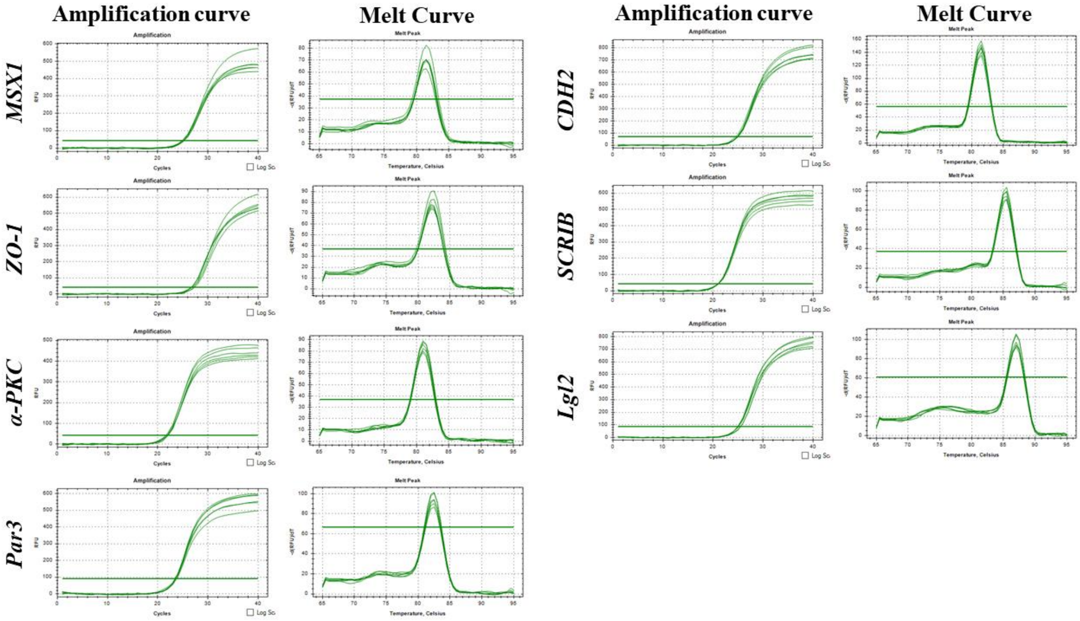

Figure S4. The negative control of F-IHC.
